# Isolation and characterization of novel *Bacillus* strains with superior probiotic potential: comparative analysis and safety evaluation

**DOI:** 10.1038/s41598-024-51823-z

**Published:** 2024-01-17

**Authors:** Mohsen Golnari, Nastaran Bahrami, Zahra Milanian, Mohammad Rabbani Khorasgani, Mohammad Ali Asadollahi, Rasoul Shafiei, Seyed Safa-Ali Fatemi

**Affiliations:** 1 Department of Biotechnology, Faculty of Biological Science and Technology, University of Isfahan, Isfahan, Iran; 2 Department of Microbiology, NourDanesh Institute of Higher Education, Meymeh, Iran; 3 Department of Microbiology, Faculty of Biological Science and Technology, University of Isfahan, Isfahan, Iran; 4 Department of Systems Biotechnology, Institute of Industrial and Environmental Biotechnology, National Institute for Genetic Engineering and Biotechnology (NIGEB), Tehran, Iran

**Keywords:** Biotechnology, Microbiology

## Abstract

Despite the current use of some *Bacillus* spp. as probiotics, looking for and introducing new efficient and safe potential probiotic strains is one of the most important topics in both microbiology and food industry. This study aimed to isolate, identify, and evaluate the probiotic characteristics and safety of some *Bacillus* spp. from natural sources. Thirty-six spore-forming, Gram-positive, and catalase-positive *Bacillus* isolates were identified in 54 samples of soil, feces and dairy products. Bacterial identification was performed using 16S rDNA sequencing. To evaluate the probiotic potential of isolates, the resistance of bacterial cells to simulated gastrointestinal tract (GIT) conditions, the presence of enterotoxin genes, their susceptibility to antibiotics, antimicrobial and hemolytic activities and biochemical profiles were investigated. The results revealed that eight sporulating *Bacillus* spp. isolates fulfilled all tested probiotic criteria. They showed a high growth rate, non-hemolytic and lecithinase activity, and resistance to simulated GIT conditions. These strains exhibited broad-spectrum antibacterial activity against pathogenic bacteria. In addition, they did not exhibit antibacterial resistance to the 12 tested antibiotics. The results of this study suggest that these isolates can be considered as candidates for functional foods and as safe additives to improve diet quality.

## Introduction

Probiotics are considered to be living and non-pathogenic microorganisms that can enhance the intestinal microbial balance in favor of beneficial microorganisms colonizing in the human intestine. They are used as functional foods and dietary supplements, alternatives to antibiotics in animal farming, growth promoters in feed supplements, and human and aquaculture prophylactics^1,2^. Previous studies have shown that dietary supplementation with *Bacillus*-based probiotics could be successfully used in poultry diets with growth-promoting benefits^3^. *Bacillus*-based probiotic products are also available for human consumption.

Bacterial probiotics mainly belong to lactic acid bacteria (LAB) and *Bifidobacteria*^4,5^. Therefore, *Bacillus* species have received limited attention compared with LAB and *Bifidobacteria*. Nevertheless, Enterogermina® (Sanofi-Aventis SpA), a probiotic product containing *B. clausii* spores was registered in 1958 as an OTC medicinal supplement in Italy. Some strains of rod-shaped, Gram-positive, catalase-positive and spore-forming Bacilli including *B. laterosporus*, *B. subtilis*, *B. clausii, B. licheniformis*, *B. cereus, B. coagulans* and *B. pumilus* have been shown to possess probiotic characteristics^6,7^.

Growth and sporulation rate, the resistance of spores to gastrointestinal tract (GIT) condition, broad antimicrobial activities against pathogenic bacteria, lack of antibiotic resistance, lack of positive hemolytic and lecithinase activity, and lack of enterotoxin genes involved in gastrointestinal disorders are the most important criteria that a microorganism must possess to be considered as a probiotic strain. *Bacillus* species offer several advantages over lactobacilli and *Bifidobacteria* as probiotics. Since these bacteria are spore formers, they can survive for a long time at room temperature in a desiccated form. In addition, their endospore structure allows them to withstand harsh conditions in the GIT. They can survive in extreme or stressful environmental conditions such as low pH, high temperatures, high bile salts, nutrient starvation and desiccation^8^. Furthermore, Bacilli exhibit high metabolic activity mainly associated with the synthesis of a vast array of antimicrobial compounds. For instance, approximately 4–5% of the *B. subtilis* genome has been devoted to the synthesis of antibiotics, enabling these bacteria to produce more than two dozen antibiotics^9^. Moreover, several *Bacillus* species are remarkably important in the industry due to their application in the industrial production of extracellular enzymes such as amylase and protease^10,11^.

Given the interesting features of *Bacillus* species as promising probiotic candidates and the limited presence of *Bacillus* probiotics in the market, this study sought to isolate favorite *Bacillus* strains from various sources including soil, feces and artisanal dairy products. Following isolation, these strains were identified, and their probiotic properties were systematically investigated and compared with those of some established commercial *Bacillus* probiotics.

## Results

### Identification of probiotic isolates

From 54 samples of dairy products, soil, livestock and poultry wastes, in total 36 spore-forming, Gram-positive and catalase-positive strains were isolated (Table 1). The PCR products of the 16S rRNA genes were analyzed using gel electrophoresis, revealing a prominent 1500 bp band. The alignment results for the most of isolates exhibited a high similarity of 98–100% in the 16S rDNA of the Bacilli. The accession numbers of the registered sequences are represented in Table 1. However, the isolates identified as *B. cereus*, which possess potential pathogenicity, and two strains with limited growth capability were excluded from further testing to ensure the safety and focus of the subsequent investigations.

**Table 1:** Identification of *Bacillus* spp. by 16S rDNA gene sequencing, the origin of isolates, and their GenBank accession numbers.

Isolate	Strain	Identity (%)	GenBank accession number	Isolation source
Khk	*B. coagulans*	98	KX261624	Soil
BUA	*B. pumilus*	100	KX270715	Turkey feces
BC	*B. safensis*	99	KX270714	Soil
GB	*B. endophyticus*	99	KX261623	Sheep feces
CF	*B. amyloliquefaciens*	99	KX270716	Milk
Ga1a	*B. pumilus*	99	KX261622	Cow feces
DF	B. cereus*	99	KX270717	Dough
G1	*B. pumilus*	99	KX270718	Sheep feces
GE1	*B. pumilus*	93	KX270719	Cow feces
GR	B. cereus*	98	KX270720	Cheese
Gsa2	*B. pumilus*	98	KX270721	Sheep feces
Ma1c	*B. toyonensis*	99	KX270724	Chicken feces
Gua	*B. licheniformis*	99	KX270722	Cow feces
Ma1b	*B. licheniformis*	99	KX270723	Chicken feces
Ma3b	*B. safensis*	99	KX270725	Chicken feces
ME	*B. amyloliquefaciens*	99	KX270726	Chicken feces
MZ	B. cereus*	99	KX270727	Yoghurt
SHE	*B. amyloliquefaciens*	99	KX270728	Camel feces
BCN	*B. subtilis*	100	KX276169	Sheep feces
SR	*B. pumilus*	100	KX276170	Traditional apple vinegar
PR	*B. subtilis*	99	KX276171	Cheese
KHG2	*B. aryabhattai*	99	KX276172	Soil
KHM1	B. cereus*	100	KX276173	Soil
GA3A	*B. pumilus*	99	KX276174	Cow feces
GA3B	B. pumilus*	100	KX276175	Cow feces
PUN	*B. siamensis*	99	KX276176	Cheese
G2a	*B. subtilis*	99	KX276177	Sheep feces
GE2b	*B. mojavensis*	100	KX276178	Cow feces
GA2	B. licheniformis*	100	KX276179	Cow feces
ME2a	*B. toyonensis*	98	KX276180	Yoghurt
MA2b	*B. licheniformis*	98	KX276181	Chicken feces
KHP3	B. subtilis*	99	KX276182	Soil
MM	*B. amyloliquefaciens*	99	KX267183	Yoghurt
GHE	*B. pumilus*	98	KX276184	Goose feces
MA1d	*B. amyloliquefaciens*	99	KX276185	Chicken feces
PTL2	B. cereus*	99	KX276186	Cheese

The sequences were deposited in NCBI GenBank and are openly accessible (See “Data availability” statement).

*These strains were excluded from further analyses.

### Hemolytic and lecithinase activity of isolates

As demonstrated in Table 2, it was found that 18 strains out of 28 strains were gamma hemolytic (non-hemolytic), five strains were alpha-hemolytic, and five strains showed beta-hemolytic activity. Also, the lecithinase test results were negative for all strains except one isolate (PR) (Table 2).

**Table 2:** Results of hemolytic activity and lecithinase test.

Isolates	Hemolytic activity	Lecithinase test
BC	⍺	–
G1	ɤ	–
GB	ɤ	–
Khk	ɤ	–
ME	ɤ	–
CF	ɤ	–
Ma1b	ɤ	–
Ga1a	ɤ	–
SHE	ɤ	–
GE1	ɤ	–
Gua	ɤ	–
BCN	β	–
Ma1c	β	–
BUA	⍺	–
Ma3b	⍺	–
Gsa2	⍺	–
GA3a	ɤ	–
ME2a	β	–
GE2b	ɤ	–
GHE	ɤ	–
MA1d	ɤ	–
G2a	ɤ	–
MA2b	β	–
Khg2	β	–
PR	ɤ	+
SR	⍺	–
MM	ɤ	–
PUN	ɤ	–

(ɤ) non-hemolytic; (⍺) hemolytic; (β) complete hemolytic.

### Probiotic characteristics

#### Resistance of the strains to simulated GIT conditions

Survival rates of the 17 isolates were evaluated under simulated gastric fluid (SGF) and simulated intestinal fluid (SIF) conditions. Based on the results, the viability of 13 strains was above 90% under GIT conditions and only 4 strains showed reduced viability of more than 1 log (Table 3).

**Table 3:** Evaluation of acid and bile salt tolerance of *Bacillus* spp. after exposure to simulated gastrointestinal condition.

Isolates	Viable cells exposed to SGF (log CFU/mL)			Viable cells exposed to the SIF (log CFU/mL)			
	0 min	30 min	60 min	0 min	60 min	120 min	180 min
Ga3a	8.1 ± 0.06	8 ± 0.04	8 ± 0.03	8.1 ± 0.04	8.1 ± 0.6	8 ± 0.07	8 ± 0.07
Ghe	8.2 ± 0.04	8 ± 0.07	8 ± 0.04	8.2 ± 0.06	8 ± 0.05	7.9 ± 0.06	7.9 ± 0.04
Ma1d	8.3 ± 0.07	8.3 ± 0.07	8.3 ± 0.05	8.7 ± 0.03	8.7 ± 0.03	8.7 ± 0.04	8.6 ± 0.05
G2a	8.9 ± 0.05	8.9 ± 0.05	8.9 ± 0.07	8.7 ± 0.05	8.7 ± 0.05	8.7 ± 0.05	8.7 ± 0.08
GE2b	8.5 ± 0.03	8.5 ± 0.04	8.5 ± 0.03	8.9 ± 0.06	8.9 ± 0.06	7.8 ± 0.09	No growth
PUN	8.3 ± 0.03	8.3 ± 0.06	8.3 ± 0.06	8.3 ± 0.03	8.3 ± 0.04	8.3 ± 0.06	8.3 ± 0.03
MM*	8.5 ± 0.05	8.5 ± 0.05	No growth	8.9 ± 0.05	8.9 ± 0.07	8.9 ± 0.06	8.9 ± 0.07
G1	8.2 ± 0.06	8.2 ± 0.06	8.1 ± 0.07	8.3 ± 0.06	8.3 ± 0.04	8.3 ± 0.04	8 ± 0.05
GB	8.8 ± 0.05	8.7 ± 0.06	8.7 ± 0.04	8.6 ± 0.02	8.6 ± 0.03	8.6 ± 0.03	8.5 ± 0.03
Khk	8.2 ± 0.06	7.9 ± 0.08	7.9 ± 0.08	8.2 ± 0.06	7.9 ± 0.05	7.9 ± 0.05	7.8 ± 0.07
ME*	8.9 ± 0.06	8 ± 0.09	No growth	8.3 ± 0.03	8 ± 0.05	7.7 ± 0.06	7.5 ± 0.06
CF*	9.4 ± 0.07	7.8 ± 0.08	No growth	8.9 ± 0.05	8.9 ± 0.05	7.7 ± 0.06	7.9 ± 0.05
Ma1b	9.9 ± 0.03	9.9 ± 0.05	9.9 ± 0.05	8.9 ± 0.06	8.9 ± 0.06	8.9 ± 0.07	8.9 ± 0.03
Ga1a	8.5 ± 0.08	8.5 ± 0.06	8.5 ± 0.08	9.4 ± 0.04	9.4 ± 0.03	9.3 ± 0.05	9.3 ± 0.06
SHE	8.9 ± 0.05	8.9 ± 0.08	8.9 ± 0.07	8.9 ± 0.03	8.9 ± 0.06	8.9 ± 0.08	8.9 ± 0.08
GE1	7.5 ± 0.05	7.5 ± 0.03	7.5 ± 0.08	8.4 ± 0.07	8.3 ± 0.05	8.3 ± 0.06	8.3 ± 0.07
Gua	10.4 ± 0.03	10.4 ± 0.06	10.4 ± 0.06	10 ± 0.04	10 ± 0.04	10 ± 0.05	10 ± 0.05

Values are representatives of mean ± S.D. (n = 3). P value < 0.05.

Results are expressed in log (CFU/mL) and experiments were repeated in three independent trials.

*P value > 0.05.

#### Antibiotic susceptibility

Antibiotic resistance of the strains was evaluated according to the European food safety authority (EFSA) and National Committee for Clinical Laboratory Standards (NCCLS). Due to the lack of a reliable interpretation document for the disc diffusion method for *Bacillus* spp., the inhibition zones lower than 12 mm were considered resistant to antibiotics as suggested by Hoa et al.^12^. Table 4 shows the results of the disc diffusion method, indicating that G2a, SHE, GHE, and GUa isolates demonstrated resistance to streptomycin. Fortunately, the resistance gene to this antibiotic is not transferable^13^. While no inhibition zone was observed for the Gala isolate in relation to chloramphenicol, the microdilution assay confirmed its susceptibility to this antibiotic. Also, the Gua isolate was resistant to erythromycin, clindamycin, chloramphenicol as well as ampicillin based on the microdilution method.

**Table 4:** Antibiotic resistance of the isolates using DDM, MIC and MBC methods.

		Isolates												
		G1	GB	Ma1b	Khk	Ga1a	SHE	GE1	Gua	PUN	G2a	MA1d	GHE	GA3a
Vancomycin	DDM	23 (S)	22 (S)	25 (S)	31 (S)	22 (S)	19 (S)	23 (S)	22 (S)	23 (S)	25.5 (S)	21 (S)	20 (S)	20 (S)
	MIC	0.25 (S)	0.25 (S)	0.25 (S)	0.25 (S)	0.25 (S)	0.25 (S)	0.25 (S)	0.25 (S)	0.25 (S)	0.25 (S)	0.25 (S)	0.25 (S)	0.25 (S)
	MBC	0.25	0.25	0.25	0.25	0.25	0.25	0.25	0.25	0.25	0.25	0.25	0.25	0.25
Erythromycin	DDM	29 (S)	19 (S)	28 (S)	33 (S)	23 (S)	26 (S)	– (R)	– (R)	32 (S)	39 (S)	29 (S)	26 (S)	22 (S)
	MIC	0.5 (S)	1 (I)	0.125 (S)	0.125 (S)	0.125 (S)	0.125 (S)	> 64 (R)	> 64 (R)	0.125 (S)	0.125 (S)	0.125 (S)	0.125 (S)	0.125 (S)
	MBC	0.5	0.25	0.25	0.125	0.5	0.25	(R)	(R)	0.125	0.125	0.125	0.125	0.125
Clindamycin	DDM	32 (S)	27 (S)	25 (S)	38 (S)	24 (S)	23 (S)	13 (S)	– (R)	23 (S)	25 (S)	30 (S)	29 (S)	16 (S)
	MIC	0.25 (S)	1 (I)	1 (I)	0.125 (S)	1 (I)	0.25 (S)	> 64 (R)	> 64 (R)	0.125 (S)	0.25 (S)	0.25 (S)	0.25 (S)	2 (I)
	MBC	0.5	4	> 4	0.125	> 4	0.5	(R)	(R)	0.25	4	2	0.25	8
Tetracycline	DDM	30 (S)	33 (S)	31 (S)	36 (S)	30 (S)	13 (S)	26 (S)	32 (S)	19 (S)	32 (S)	23 (S)	31 (S)	30 (S)
	MIC	0.25 (S)	0.5 (S)	1 (S)	1 (S)	2 (S)	16 (R)	4 (S)	1 (S)	4 (S)	8 (I)	8 (I)	1 (S)	0.25 (S)
	MBC	0.25	0.5	2	2	4	16	8	2	8	8	16	2	> 1
Chloramphenicol	DDM	24 (S)	17 (S)	18 (S)	32 (S)	– (R)	23 (S)	19 (S)	15 (I)	32 (S)	29 (S)	30 (S)	21 (S)	19 (S)
	MIC	8 (S)	8 (S)	4 (S)	4 (S)	8 (S)	1 (S)	8 (S)	32 (R)	2 (S)	1 (S)	2 (S)	8 (S)	8 (S)
	MBC	8	8	> 16	16	32	1	16	32	4	2	4	8	16
Ampicillin	DDM	31 (S)	32 (S)	29 (S)	42 (S)	25 (S)	16 (S)	21 (S)	18 (S)	32 (S)	31 (S)	29 (S)	29 (S)	28 (S)
	MIC	0.125 (S)	0.125 (S)	0.125 (S)	0.125 (S)	0.125 (S)	0.125 (S)	0.125 (S)	0.5 (R)	0.125 (S)	0.125 (S)	0.125 (S)	0.125 (S)	0.125 (S)
	MBC	0.125	0.125	0.125	0.25	0.25	0.25	> 0.5	0.5	0.25	0.125	0.125	0.125	> 0.5
Kanamycin	DDM	18 (S)	24 (S)	20 (S)	34 (S)	21 (S)	18 (S)	26 (S)	16 (S)	24 (S)	21 (S)	24 (S)	16 (S)	18 (S)
Penicillin	DDM	34 (S)	28 (S)	41 (S)	44 (S)	31 (S)	18 (S)	20 (S)	22 (S)	31 (S)	32 (S)	32 (S)	31 (S)	31 (S)
Streptomycin	DDM	14 (S)	23 (S)	18 (S)	29 (S)	17 (S)	– (R)	18 (S)	10 (R)	23 (S)	11 (R)	21 (S)	11 (R)	12 (S)
Gentamicin	DDM	18 (S)	23 (S)	20 (S)	40 (S)	17 (S)	18 (S)	22 (S)	16 (S)	25 (S)	20 (S)	23 (S)	15 (S)	17 (S)

*S* susceptible, *R* resistant represented in brackets.

#### Antimicrobial activity

The ability to inhibit the growth of pathogens is a favorable characteristic of probiotic bacteria. Based on the well-diffusion method, the diameters of the growth inhibition zones of the pathogens treated with the most isolated strains were more than 10 mm (Table 5). The GE1 strain inhibited a broad range of pathogenic bacteria, and the SHE strain displayed the greatest effect on MRSA with a 23 mm diameter inhibition zone. However, none of the isolates exhibited antibacterial activity against the *ESBL E. coli*.

**Table 5:** Antibacterial activity of cell-free supernatant of the isolated strains against some pathogenic bacteria.

Pathogenic bacteria	The inhibition zone diameter (mm) caused by the isolates												
	G1	GB	Ma1b	Khk	Ga1a	SHE	GE1	Gua	PUN	G2a	Ma1d	GHE	Ga3a
*K. pneumoniae*	NO	NO	NO	17 ± 0.6	NO	NO	10 ± 0.5	17 ± 0.5	NO	20 ± 0.7	11 ± 0.8	NO	20 ± 0.9
*MRSA*	NO	NO	NO	NO	NO	22 ± 0.5	11 ± 0.4	NO	NO	NO	NO	9 ± 0.5	NO
*E. faecium*	NO	NO	NO	16 ± 0.5	12 ± 0.7	15 ± 0.5	11 ± 0.5	13 ± 0.3	11 ± 0.5	15 ± 1	16 ± 0.7	11 ± 0.7	18 ± 0.8
*VRE*	12 ± 0.5	NO	NO	NO	NO	15 ± 0.8	15 ± 0.4	NO	NO	NO	21 ± 1	NO	10 ± 0.6
*A. baumannii*	NO	NO	NO	NO	NO	13 ± 0.5	NO	NO	NO	NO	NO	NO	NO
*S. typhimurium* ATCC1609	20 ± 1	15 ± 0.6	NO	NO	NO	NO	12 ± 0.6	NO	13 ± 0.5	NO	18 ± 1	NO	NO
*ESBL E. coli*	NO	NO	NO	NO	NO	NO	NO	NO	NO	NO	NO	NO	NO
*S. sonnei* ATCC9290	15 ± 1	9 ± 0.5	9 ± 0.5	16 ± 1	18 ± 1	NO	11 ± 0.5	16 ± 1	19 ± 0.5	8 ± 0.5	16 ± 1	12 ± 0.4	10 ± 0.4
*L. monocytogenes* ATCC7624	NO	12 ± 0.6	NO	NO	NO	8 ± 0.8	16 ± 1	NO	10 ± 0.2	13 ± 1	11 ± 0.5	NO	NO
*S. aureus* ATCC25923	NO	NO	NO	NO	14 ± 2	NO	12 ± 0.5	11 ± 0.5	15 ± 0.7	11 ± 0.5	13 ± 0.7	16 ± 0.4	8 ± 0.5
*S. pyogenes*	11 ± 0.3	17 ± 0.6	14 ± 0.5	NO	17 ± 0.5	NO	NO	22 ± 0.7	6 ± 0.2	NO	NO	NO	NO
*E. coli* ATCC25922	NO	14 ± 0.6	NO	NO	9 ± 0.5	NO	10 ± 0.5	NO	11 ± 0.6	NO	NO	NO	NO

Values are representatives of mean ± S.D., (n = 3). )NO: indicates not observed any inhibition zone).

#### Enterotoxins and other potential virulence factors

*Nhe*, *hbl* and *bceT* genes were detected using PCR to distinguish virulent strains (Table 6). To evaluate whether these enterotoxin genes are essentially involved in hemolytic activity, ME2a, MA2b, MA1c and Khg2, as β-hemolytic isolates, were investigated for the presence of enterotoxin genes by PCR test. The results showed that ME2a and MA1c possess all enterotoxin genes and were considered as the positive controls for the PCR test of other samples. In four of the γ-hemolytic isolates, namely, GA3a, GHE, SHE and Gua, the presence of enterotoxin genes (*bceT*, *hbl* and *nhe*) was identified.

**Table 6:** Presence of enterotoxin genes in the isolates.

Isolates	Genes							
	*hblA*	*hblB*	*hblC*	*hblD*	*nheA*	*nheB*	*nheC*	*bceT*
G1	–	–	–	–	–	–	–	–
GB	–	–	–	–	–	–	–	–
Khk	–	–	–	–	–	–	–	–
Ma1b	–	–	–	–	–	–	–	–
Ga1a	–	–	–	–	–	–	–	–
GE1	–	–	–	–	–	–	–	–
SHE	+	+	+	–	–	–	–	–
Gua	–	–	–	+	–	–	+	–
PUN	–	–	–	–	–	–	–	–
G2a	–	–	–	–	–	–	–	–
Ma1d	–	–	–	–	–	–	–	–
GHE	–	–	–	–	+	–	–	+
Ga3a	–	–	–	–	+	+	+	–
Ma1c	+	+	+	+	+	+	+	+
ME2a	+	+	+	+	+	+	+	+

+ : positive, –: negative.

#### Biochemical tests

Various carbohydrates were tested to be used as sole carbon sources by the isolated strains and the results are presented in Table 7. It was shown that all strains produced acid from xylose, trehalose, and glucose. None of the strains could ferment lactose, sorbitol, melibiose and raffinose. Only GB isolate fermented rhamnose. Mannitol and fructose were fermented by three and five of the isolates, respectively.

**Table 7:** Utilization of different carbon sources by the isolated *Bacillus* spp.

Isolates	Sugar											
	Glucose	Fructose	Lactose	Trehalose	Rhamnose	Sorbitol	Melibiose	Raffinose	Mannitol	Sucrose	Starch	Xylose
GE1	+	+	–	+	–	–	–	–	–	+	+	+
SHE	+	+	–	+	–	–	–	–	–	+	+	+
G1	+	–	–	+	–	–	–	–	+	–	–	+
GB	+	–	–	+	+	–	–	–	+	+	+	+
Khk	+	–	–	+	–	–	–	–	–	+	+	+
Ga1a	+	+	–	+	–	–	–	–	+	+	+	+
Ma1b	+	+	–	+	–	–	–	–	–	+	+	+
PUN	+	–	–	+	–	–	–	–	–	+	+	+
G2a	+	+	–	+	–	–	–	–	–	+	+	+
MA1d	+	–	–	+	–	–	–	–	–	+	+	+

+ : positive, –: negative.

Table 8 presents the results of biochemical tests and the growth characteristics of the strains under harsh conditions. It can be concluded that most isolates were able to survive at high temperatures (55 °C). Notably, the G2a isolate grew even at 65 °C. However, both SHE and Ma1b isolates were unable to withstand 6.5% NaCl. As for resistance to low pH environments, the PUN, G2a, and MA1d isolates were able to survive at pH values ranging from 4.0 to 5.0.

**Table 8:** Biochemical properties of the isolated strains and their ability to grow in some harsh conditions.

Isolates	Test									
	Starch hydrolysis	VP test	Nitrate reduction	citrate utilization	Growth in temperature		Growth in pH		Growth in salt concentrations	
					55° C	65° C	4	5	% 4	% 6.5
GE1	–	+	–	+	+	–	–	–	+	+
SHE	+	+	+	–	–	–	–	+	+	–
G1	–	–	–	+	+	–	–	–	+	+
GB	–	–	–	–	+	–	–	+	+	+
Khk	–	+	–	–	+	–	–	+	+	+
Ga1a	–	+	–	+	+	–	–	–	+	+
Ma1b	+	+	+	+	–	–	–	+	–	–
PUN	+	–	+	–	+	–	+	+	+	+
G2a	+	–	+	+	–	+	+	+	+	+
MA1d	+	+	+	+	+	–	+	+	+	+

+ : positive, –: negative.

## Discussion

### Isolation of *Bacillus* strains

*Bacillus* species are ubiquitous in nature and can be isolated from a variety of ecological habitats. In this study, several *Bacillus* strains were isolated from diverse sources, including soil, feces, and artisanal dairy products. While soil serves as a prominent environmental niche for numerous Bacillus species, *Bacillus* strains displaying promising probiotic properties can also be found in animal feces^14^ and artisanal dairy products^15^. Some of isolated *Bacillus* strains such as *B. amyloliquefaciens*^16^ and *B. siamensis*^17^, have previously been isolated and identified in similar researches. Sen et al. isolated and identified *B. coagulans* strains with probiotic potential from soil samples containing dry animal waste. In our investigation, four strains of *B. cereus* were isolated from dairy samples, which aligns with findings of Montanhini et al. who also isolated 23 *B. cereus* strains from similar samples^18^. Eight *B. pumilus* strains were isolated from livestock and poultry wastes. Guo et al. isolated this strain with probiotic properties from the fresh waste of Tibetan sheep^19^. Also, five *B. amyloliquefaciens* strains were isolated from camel and chicken feces and dairy products, while this strain was recently isolated from soil, fruits, and cereals^20–22^. In a recent study, Daneshazari et al. isolated *Bacillus* strains from camel milk with probiotic potentials^23^ further contributing to the growing body of knowledge on beneficial *Bacillus* strains.Furthermore, two strains of *B. safensis* were isolated from soil and poultry waste samples. Satomi et al. have also isolated 30 strains of *Bacillus* spp. from spacecraft and assembly-facility surfaces from which 13 strains were identified as *B. safensis*^24^. Raja and Omine isolated *B. safensis* MS11 strain from forest soil samples of Mongolia^25^.

Additionally, one *B. toyonensis* strain was identified from poultry waste samples. There are few reports on the isolation of this bacterium. However, recently Okaiyeto et al. have purified *B. toyonensis* from South African sea sediments^26^. In Japan, this strain is used in a probiotic product named Toyocerin^27^.

### Probiotic characterization of isolates

#### Hemolytic activity of isolates

Non-hemolytic (ɤ-hemolytic) strains are generally considered safe for their hosts, while strains with hemolytic activity are considered pathogen. Microorganisms exhibiting hemolytic activity can disrupt red blood cells, leading to the release of hemoglobin. This activity is often associated with the production of hemolysins, which can have cytolytic effects on host cells, and decreasing available hemoglobin content as a Fe source^28,29^. On the other hand, beta hemolytic activity indicates the presence of cytotoxic phospholipase in microorganisms^6^.

Investigation of the hemolytic activity of 48 *Bacillus* strains isolated from 50 probiotic products collected from South Korea, Australia, and China revealed that 58% of the isolates displayed hemolytic activity^30^. Therefore, strains displaying hemolytic activities were excluded from further assessment, as they may pose a risk to the host's health.

#### Resistance to simulated GIT conditions

The ability to tolerate high acid concentrations in the stomach is a relevant criterion for selecting an appropriate probiotic product^31^. Some isolated strains demonstrated remarkable resistance to the GIT conditions. Interestingly, in certain cases, these isolated strains exhibited even better viability than commercially available strains like Subtyl^32^, which indicates their potential as robust candidates for probiotic applications. This increased tolerance to acidic environments enhances their chances of survival and exerting their beneficial effects in the GIT, making them promising candidates for further probiotic investigations.

The microaerophilic conditions prevailing in the digestive tract highlights the importance of probiotic strains' ability to thrive in such environments. In our study, we observed that after 24 h of incubation in microaerophilic conditions, all samples exhibited robust growth. Since gastrointestinal juice imposes stressful conditions against bacteria, tolerance to gastrointestinal juice is a critical property sought in probiotic bacteria^33^. Sharma et al. suggested that an appropriate probiotic strain should withstand at least pH 3.0^34^, while Hong et al. reported that *Bacillus* strains exhibit different resistances to gastric acid and not all are resistant to these conditions^35^. The disparity in resistance might be attributed to the activation of spores exposed to acids and the subsequent destruction of regenerative cells in the acidic environment of the stomach^13,32^. Understanding these mechanisms is vital for identifying probiotic strains with the highest likelihood of surviving in the GIT.

Probiotic bacteria must possess the property to pass through the small intestine successfully^36^. However, bile salt and pancreatic enzymes of the small intestine can be detrimental to bacterial cells^37^. The isolated spore-forming *Bacillus* spp. showed proper resistance against pepsin and pancreatin, two of the gastrointestinal enzymes. These results suggest that many of the strains could reach the colon without significantly being affected by gastrointestinal conditions. In a recent study, it was shown that the *B. coagulans* population decreased only by 0.64 log under semi-intestinal conditions. It could be concluded that using *Bacillus* spp. for probiotic consumption offers functional benefits because of their resistance to food processing temperature, storage, and GIT environment^38^. Their ability to withstand these challenges suggests their potential as robust probiotic candidates with a high likelihood of providing health benefits to the host.

#### Antibiotic resistance of isolates

Although no concerning antibiotic resistance was observed regarding the *Bacillus* spp. isolated in this study, the results of some previous studies already conducted on commercial probiotic products, such as HU36, Natto and PY79, showed the resistance of HU36 strain to clindamycin. The PY79 strain was also resistant to streptomycin and tetracycline. Likewise, the Natto strain showed resistance to streptomycin^35^. Furthermore, a study on commercial probiotic strains, including Enterogermina and Biosubtyl revealed Enterogermina resistance to penicillin, erythromycin, lincomycin, rifampin, neomycin, and chloramphenicol antibiotics^39^.

#### Antibacterial activity of isolates

Antibiotics are among the most important pharmaceuticals, having saved millions of lives since their introduction to the market. However, the excessive use of antibiotics has raised concerns about the development of antibiotic-resistant pathogens and reduced efficacy, posing a significant threat to human life. While ongoing efforts to discover novel antibiotics persist, the consumption of probiotics harboring antimicrobial activity could serve as a potential mitigation strategy for this problem.

As shown in Table 5, several isolates displayed antimicrobial activity. Notably, the GE1 isolate exhibited a broad-spectrum antimicrobial effect against 9 out of the 12 of the examined antibiotics, including MRSA, thereby suggesting this isolate a promising probiotic with potent antimicrobial properties.

The antibacterial activity of the isolates might be due to the production of bacteriocin-like agents and also peptide and non-peptide antibiotics released by bacterial cells^40^. In recent studies, it was shown that *Bacillus* strains produce a broad range of antibacterial agents including peptide and lipopeptide compounds against Gram-positive and Gram-negative pathogens^41–43^. Also, the presence of secondary metabolites with antimicrobial potentials, such as bacteriocin, hydrogen peroxide, lactic acid and propionic acid, in bacterial culture supernatant has previously been reported^44^. Together with the competitive exclusion mechanism, where probiotics compete with harmful bacteria for adhesive receptors and nutrients, these metabolites can effectively destroy and prevent the colonization of pathogens in the body^45^.

#### Presence of enterotoxin encoding genes

Although a few numbers of *Bacillus* spp. have been shown to produce toxins, some *Bacillus* spp. including *B. endophyticus*, *B. subtilis*, *B. amyloliquefaciens*, *B. pumilus* and *B. licheniformis* have been approved as generally recognized as safe (GRAS) probiotic bacteria^43,46^. Moreover, endospores of certain *Bacillus* spp. have been studied as Direct Feed Microbials (DFM), and the findings have confirmed their safety and reliability as prophylactic agents in reducing GI disease in livestock and humans^47,48^. The results of the current study are in good agreement with some similar reports^43,46,49,50^.

Evaluation of DNA showed that the strains carrying the genes coding for two enterotoxins hemolysin (Hbl) and (Nhe) are non-hemolytic enterotoxins^51,52^. While conclusion about protein function or malfunction solely based on the results of DNA research is not sufficient, this hypothesis still exists that genes encoding for HH and Nhe enterotoxins may not function in some *Bacillus* strains^53^. Results of a study on the possible pathogenicity of some commercial probiotic strains including Subtyl, Bactisubtil and Biosubtyl^DL^ revealed hemolytic and lecithinase activity in these products and the presence of *hbl* and *nhe* enterotoxin genes in these strains was confirmed^32^.

#### Physiological characterization of isolates

Some isolates of the present study showed diverse sugar fermentation profiles based on Bergey’s manual of systematic bacteriology book^54^. One of the main reasons for the discrepancy between the biochemical tests can be attributed to the loss or gain of transferable genes. Since some genes needed for the fermentation of sugars are encoded by plasmids, variation in plasmid content may result in metabolic inconsistencies^55^.

As expected, all the isolates were able to ferment glucose as the sole carbon source. However, none of the isolates were able to utilize lactose (Table 7). Except for PUN, other strains in Table 7 were isolated from soil or animal feces. These environments typically do not provide a consistent source of lactose, and *Bacillus* species from these habitats may not have evolved mechanisms for efficient lactose utilization.

## Conclusion

In this study, we isolated 36 *Bacillus* species from soil, animal feces, and artisanal dairy products samples collected from various locations in Iran. After conducting a comprehensive assessment of the probiotic properties of the isolates based on available studies, we identified eight *Bacillus* strains that met the probiotic criteria. These strains included G2a (*B*. *subtilis* strain esf-G2; KX276177), MA1d (*B. amyloliquefaciens* strain ard-MA1D; KX276185), Khk (*B*. *coagulanse* strain NBRC-12583; KX261624), GB (*B*. *endophyticus* strain 2DT; KX261623), G1 (*B. pumilus* strain esf G1; KX270718), Ga1a (*B*. *pumilus* strain NBRC-12092; KX261622), Ma1b (*B*. *licheniformis* strain ard MA1B; KX270723) and PUN (*B*. *siamensis* strain ard-PUN; KX276176). The results of this study suggest that these isolates, especially G2a, Ga1a, G1and Khk, exhibit significant potential for utilization in several sectors, including animal feed additives, promoting gut health, food preservatives, and antibiotic alternatives. However, further in vivo analyses are required to unlock the full spectrum of potential applications for these isolates. Comprehensive in vivo assessments will not only validate the findings but also enhance and broaden the potential applications of these isolates across diverse fields. Moreover, considering the prevalence of probiotic products in the market primarily consisting of LAB and *Bifidobacteria*, future investigation should focus on exploring the synergistic or antagonistic interactions of our isolated *Bacillus* strains with these well-established probiotic groups.

## Materials and methods

### Sample collection and isolation of strains

A total of 54 samples, including soil, animal feces, and artisanal dairy products were taken from Isfahan and Ardabil provinces in Iran. In summary, the collection comprised 5 soil samples (3 from agricultural setting and 2 from greenhouses), 18 samples from animal feces (6 from chickens, 5 from cows, 4 from sheep, 1 from turkeys, 1 from gooses, and 1from camels), and 31 samples from a diverse array of artisanal dairy products. The samples were placed in sterile vials, promptly chilled on ice, and transported to the laboratory.

One gram of each solid or 1 mL of each liquid sample was added to 10 mL of sodium citrate 2% (w/v) and shaken for 4 h. The samples were then placed in a water bath for 15 min at 80 °C to kill vegetative bacteria. Next, 1 mL of the treated samples was inoculated into 10 mL of MRS broth culture medium and incubated with shaking at 37 °C for 24 h. 100 μL of the grown cells were plated on MRS agar and incubated at 37 °C. Colonies with different morphologies were selected and subcultured. *Bacillus* colonies were screened by Gram staining, spore staining using Schaeffer-Fulton method^56^, and catalase test^57^. Finally, the pure Gram-positive, catalase-positive, and spore-forming isolates were stored as glycerol stocks at − 20 and − 80 °C until further use.

### Hemolytic and lecithinase activity

Hemolytic activity of the isolates was assayed by culturing the strains on the blood agar medium, followed by the incubation at 30 °C for 24 to 48 hours^58^. In addition, isolates were cultured on the egg yolk agar and incubated at 37 °C for 24 h to assess their lecithinase (phospholipolytic) activity^59^.

### Molecular identification of the isolate

Molecular identification of the isolates was done by PCR amplification of the corresponding 16s rRNA genes followed by their sequencing. Briefly, the genomic DNA of the pure isolates was extracted using genomic DNA extraction kits (Tali Gene Pars, Iran), and PCR was done using 27F (5′-AGAGTTTGATCCTGGCTCAC-3′) and 1492R (5’-CGGTTACCTTGTTACGACTT-3′) primers as forward and reverse universal 16S rDNA primers, respectively^60^. After gel purification, the PCR products were sequenced (Gene Fanavaran, Tehran, Iran) and analyzed by the CLC Main Workbench and Bio Edit software^61^. The National Center for Biotechnology Information (NCBI) database and the BLAST search tool were used to identify the genus and species of the bacteria.

### Evaluation of the probiotic characteristics of the isolates

#### Resistance of spores and vegetative cells to simulated GIT conditions

The acidic liquid of the stomach was simulated by adding 1 mg/mL pepsin to the sterile normal saline (0.85%) at pH 2. The bacterial suspension (0.5 McFarland) was then exposed to this solution and incubated at 37 °C. After 3, 30 and 60 min of incubation, 100 µL of each treated spore solution was inoculated into Tryptic Soy Agar (TSA) culture medium and incubated at 37 °C for 24 hours^32^.

The intestine condition was simulated by adding 1 mg/mL pancreatin and 0.2% of bile salts into the sterile isotonic buffer (pH 7.4), followed by exposing the bacterial cells to this condition. After 3, 60, 120 and 180 min, the resistance of the bacterial cells to these conditions was evaluated.

#### Antibiotic resistance of the isolates

To assess the antibiotic resistance of the isolates, the disc diffusion method (DDM) and Micro dilution methods were applied^62^. The sensitivities of the bacteria to antibiotics were interpreted as sensitive (S), intermediate (I), and resistant (R) based on the Clinical & Laboratory Standards Institute (CLSI) guidelines^63^. The Antibiotic discs used were chloramphenicol (30 µg/disc), gentamicin (10 µg/disc), erythromycin (15 µg/disc), clindamycin (2 µg/disc), kanamycin (30 µg/disc), vancomycin (30 µg/disc), penicillin (10 IU/disc), streptomycin (10 µg/disc), tetracycline (30 µg/disc) and ampicillin (10 µg/disc) that were purchased from MAST Group (Merseyside, UK). To evaluate the minimum inhibitory concentrations (MICs) of these ten antibiotics, different amounts of each antibiotic were diluted in broth medium to prepare the following concentrations: 0.5 to 256 μg/mL (chloramphenicol), 0.125 to 64 μg/mL (clindamycin, erythromycin, and ampicillin) and 0.25 to 128 μg/mL (vancomycin, tetracycline). The strains treated with these antibiotics were incubated at 35 °C for 24 h. The minimum bactericidal concentrations (MBCs) were determined as described by CLSI^63^.

#### Antimicrobial activities of the isolated strains

Antimicrobial activities of the isolated strains were assessed by spotting and agar well diffusion method against several pathogens including *Salmonella typhimurium* PTCC1609 (Persian Type Culture Collection), *Escherichia coli* ATCC11775, *Listeria monocytogenes* ATCC13932, *Shigella sonnei* ATCC9290, *Staphylococcus aureus* ATCC33591, *Klebsiella pneumoniae*, *Enterococcus faecium*, *Acinetobacter baumannii*, *Streptococcus pyogenes*, MRSA, VRE, and Extended-Spectrum Beta-Lactamase (ESBL)-producing *E. coli* provided by Alzahra hospital (Isfahan, Iran). To assess the antimicrobial activity through the agar well diffusion method, 10^6^ CFU/mL of the pathogenic bacteria suspensions were prepared and cultured on the Mueller–Hinton Agar (MHA). Wells with 0.5 cm diameter were made on MHA plates using a sterile Pasteur pipette. Two milliliters of *Bacillus* isolate medium was centrifuged at 9000×*g*, and 4 °C for 10 min^5^ and the supernatant was filtered. Next, 60 µL of the cell-free supernatant of each bacterial culture was poured into the wells and the plates were incubated at 37 °C. The diameter of the growth inhibition zoon appeared around the wells was recorded after 24 h of incubation^64–66^.

#### Presence of genes encoding virulence factors

The presence of enterotoxin genes, which are responsible for pathogenicity and food toxicity in *Bacillus* spp. was investigated. The presence of common enterotoxins coding genes in *Bacillus* bacteria including *nhe*, *hbl*, *cytk*, and *bceT*. *nhe*, *hbl*, and *bceT* was examined by multiplex PCR method using the corresponding primers for each gene as described in the literature^67–69^. A positive control and a negative control were included in the experiment.

#### Growth in microaerophilic condition

The ability of the isolates to grow in microaerophilic conditions was investigated by culturing on the Luria Bertani (LB) agar culture medium followed by incubating anaerobically at 37 °C for 24 hours^70^.

#### Biochemical tests

The isolates were also characterized with several biochemical tests including the starch hydrolysis, Voges Proskauer test, citrate test, carbohydrate fermentation and production of acid test, NaCl tolerance test, growth at high temperatures, and acid resistance test as described by Vos et al.^54^.

### Statistical analysis

Data were statistically analyzed with a significance level set at p < 0.05 using GraphPad Prism version 9.5.1.733 and one-way analysis of variance (ANOVA) was performed to identify significant differences between the means.

## Data Availability

The datasets generated and/or analysed during the current study are available in the GenBank repository, under the accession numbers stated in Table 1.
